# Combined Effects of Phytochemicals and Exercise on Fatty Acid Oxidation

**DOI:** 10.20463/jenb.2016.0053

**Published:** 2016-12-31

**Authors:** Jong-Hee Kim, Yoonjung Park

**Affiliations:** 1 Department of Physical Education, Hanyang University, Seoul Republic of Korea; 2 Department of Health and Human Performance, University of Houston, Houston U.S.A.

**Keywords:** Phytochemicals, Maximal fatty acid oxidation rate, Physical performance

## Abstract

**[Purpose]:**

The purpose of this review is to discuss current views regarding the acute effects of phytochemicals, exercise, and exercise plus phytochemicals on fatty acid oxidation.

**[Methods]:**

Data acquired from human and animal studies were comprehensively assessed to determine the single and combined effects of phytochemicals and exercise on fatty acid oxidation. In addition, underlying mechanisms associated with those conditions that may contribute to the regulation of fat metabolism are discussed.

**[Results]:**

Although not all phytochemicals are effective at increasing fatty acid oxidation, some significantly improve the rate of fatty acid oxidation at rest. In addition, dietary supplementation of p-synephrine, catechins, or anthocyanins in combination with moderately intense exercise has the additive effect of increasing fatty acid oxidation, but not total energy expenditure during exercise.

**[Conclusion]:**

The data reported from current reviewed studies suggest positive outcomes regarding facilitation of fatty acid oxidation from the combined effects of certain phytochemicals with exercise. Those data provide new insight for developing a strategy to boost fat loss and control weight in obese patients.

## INTRODUCTION

Effective management of body weight and fat mass has clinical importance because it is closely associated with health and physical performance. Obesity, resulting from abnormal or excessive fat accumulation, alters health and is becoming a major public health concern. Dietary supplementation with phytochemicals as anti-obesity agents can be an effective strategy for reducing body weight and adipose tissue mass in humans and animals^1^^-^^3^. However, the underlying mechanisms responsible for those conditions are still unknown. Some possible explanations include reduced fat absorption, inhibition of pre-adipocyte differentiation and proliferation, and stimulation of lipolysis and apoptosis of existing adipocytes^1^^, ^^4^^-^^6^. Recent human studies suggest that underlying mechanisms associated with the anti-obesity effects of phytochemicals may be partly attributable to increased fatty acid oxidation^7^^, ^^8^.

Diminished body fat through increased fat burning or fatty acid oxidation is one of the most beneficial aspects of the exercise^9^. Fatty acid oxidation during exercise varies and depends strongly on exercise intensity. Mild-to-moderate exercise increases fatty acid oxidation by 5- to 10-fold above the resting levels^10^^, ^^11^. When exercise intensity is increased further, fatty acid oxidation peaks at approximately 45–65% of maximal oxygen uptake and then gradually declines^12^^-^^14^. It is reasonable for obese or overweight people to exercise at those ranges of intensity because effective fat burning is a primary goal of treating or preventing obesity.

Given the important role of phytochemicals and exercise as anti- obesity agents or fat burners, it is imperative to determine whether the combination of phytochemicals and exercise provides an additive effect on fatty acid oxidation. To the best of our knowledge, this is the first review summarizing current perspectives available in the literature regarding the effects of phytochemicals in the form of acute dietary supplements, exercise at different intensities, and exercise plus phytochemicals on fatty acid oxidation. In this review, we also discuss potential underlying mechanisms associated with phytochemicals and exercise that may be responsible for regulating fatty acid oxidation.

## Acute Effects of Phytochemical Supplementation on Fatty Acid Oxidation

Phytochemicals are bioactive chemical compounds without energy value. These compounds are derived from plants such as fruits, beans, vegetables, and grains. Emerging reports have suggested that phytochemical-based dietary supplements may reduce adipose tissue growth, differentiation of pre-adipocytes, and appetite, as well as promote lipolysis and fatty acid oxidation, thereby facilitating weight loss and preventing obesity^3^^, ^^15^. In addition, phytochemicals have other benefits including antioxidant, anti-inflammatory, anti-aging, anti- proliferative, and anti-carcinogenic properties; they are also deemed safe and do not cause adverse health issues^15^^-^^17^.

Phytochemicals are classified into polyphenols, terpenoids, organosulfurs, and phytosterols^6^. Among the phytochemicals, polyphenols are the most effective for regulating fat metabolism by stimulating lipolysis and inducing fatty acid oxidation through multistep reactions in the mitochondria and peroxisomes^18^^, ^^19^. Polyphenols are the most diverse phytochemical family and are further classified into simple phenolic acids, stilbenes, curcuminoids, chalcones, lignans, flavonoids, and isoflavones on the basis of their chemical structure, number of phenolic rings, and biological functions^6^.

Catechins, the predominant form of phenolic acid compounds, are suggested to be a major ingredient for effective fat loss and body weight control^20^^-^^22^. Catechins are primarily contained in green tea extract. There are four major catechin forms: epigallocatechin gallate (EGCG), epigallocatechin (EGC), epicatechin gallate (ECG), and epicatechin (EC), all of which have been shown to prevent cardiovascular diseases, diabetes, cancer, and neurodegenerative diseases^23^. Among the catechins, EGCG is the most abundant and biologically active compound, comprising approximately 50% of the catechin content of green tea^24^.

Green tea catechins have been reported to increase the fat burning index using indirect calorimetry methodology, as indicated by a reduction in the respiratory exchange ratio (RER)^7^^, ^^8^^, ^^25^^-^^29^. Especially, ingestion of green tea catechins in the form of EGCG for one to three days was effective at increasing the fat burning index. Dulloo and colleagues^7^ found that supplementing with green tea extract for 24 h increases resting fatty acid oxidation by 9.9%. Moreover, Rumpler et al.^8^ showed that intake of catechin-rich oolong tea for three days increases fatty acid oxidation by 12%. In agreement with those findings, Gahreman et al.^29^ found that fatty acid oxidation is significantly increased after supplementing with catechin-rich green tea extract containing 187.5 mg polyphenols and 125 mg EGCG for one day. Caffeine constitutes 3-5% green tea extract; however, excess weight in individuals may mask its effects. A recent study demonstrated that ingestion of catechin-caffeine mixtures, but not caffeine alone, increases fatty acid oxidation, suggesting that green tea catechins increase fat burning independent of caffeine content^20^.

Anthocyanins are polyphenol flavonoids that are well-known for their antioxidant effects^30^. Anthocyanins are water-soluble pigments found in red wine, certain cereals, red/purplish fruits, and vegetables such as cabbage, grapes, apples, and beets^31^. Anthocyanins have also been shown to have anti-convulsant, anti-carcinogenic, anti-diabetic, and anti-inflammatory effects^15^^, ^^32^^, ^^33^. In addition, anthocyanin- based supplements reduce adipose tissue growth and preadipocyte differentiation, while increasing lipolysis, fat mobilization, and fatty acid oxidation (34-39); however, the reported data are from animal studies, with no explicit mechanistic evidence. Fortunately, a recent study showed that anthocyanins upregulate AMP-activated protein kinase (AMPK) and downregulate carnitine palmiyoyltranferase-1 (CPT-1), which are key molecules in the regulation of fatty acid oxidation pathways^40^.

Synephrine is a phenolic acid polyphenol that is naturally present in bitter orange and is derived from the immature fruit of *Citrus aurantium*^41^^-^^43^. *p*-synephrine is primarily found in the protoalkaloid form and is widely used as an alternative to ephedra for weight control^44^. Previous studies have demonstrated that acute and chronic ingestion of *p*-synephrine enhances resting energy expenditure, lipolysis, and breakdown of fat during rest^45^^, ^^46^. *P*-synephrine intake is associated with beta-adrenergic receptor activation, resulting in thermogenic effects^47^; however, a recent study was not able to replicate those findings when supplementing with a high dose of *p*-synephrine (3 mg/ kg, one time), which did not change energy expenditure and substrate metabolism, including fatty acid oxidation at rest^48^. The reason for this discrepancy is currently unknown; however, differences in dosages and duration of *p*-synephrine treatment, along with subjects (e.g., obese vs. non-obese, woman vs. man, young vs. old) are possible explanations. Additional, well-controlled investigations are required to define the impact of *p*-synephrine supplementation on fat metabolism.

Of all the polyphenols, resveratrol is a major stilbene compound found in red grapes, apples, peanuts, blueberries, and cranberries that has been reported to reduce adipogenesis and preadipocytes by down-regulating adipocyte- specific gene expression^49^. In addition, resveratrol has been proposed as an anti-obesity and hypolipidemic agent that increases fatty acid oxidation through upregulation of AMPK and PGC-1α^50^^, ^^51^ and downregulation of PPAR-γ, CCAAT-enhancer-binding protein (C/EBPα), and sterol regulatory element binding protein 1c (SREBP- 1c)^52^. However, it is unknown if the reduction in adipogenesis and preadipocytes as well as regulation of fatty acid oxidation observed with resveratrol will be replicated in clinical trials because most studies testing its efficacy have been undertaken in pre-clinical or animal settings.

## Acute Effects of Exercise on Fatty Acid Oxidation

Exercise intensity is a major factor that affects the fraction or rate of fatty acid oxidation^12^^, ^^53^. The fraction of fatty acid oxidation is increased during low to moderately intense exercise and progressively decreases when the exercise intensity is further increased^54^. Meanwhile, the fraction of energy supply derived from carbohydrate oxidation is augmented with increasing exercise intensity. The rate of energy obtained from fatty acid oxidation is progressively increased with increasing exercise duration. However, muscle glycogen breakdown and carbohydrate oxidation are reduced when exercise is continued. Therefore, prolonged exercise at moderate intensity is generally recommended for fat burning and weight loss.

Regulation of fatty acid oxidation in response to exercise intensity is highly dependent on five major systemic processes^14^ : 1) breakdown of triglycerides into one glycerol and three fatty acids, 2) breakdown of intramuscular triglycerides, 3) delivery of fatty acids to exercising muscles, 4) movement of fatty acids across the muscle membrane, and 5) movement of fatty acids across the mitochondrial membrane.

It has been demonstrated that the increased availability of plasma fatty acids during low to moderately intense exercise is primarily caused by an increase in lipolysis of triglycerides stored in adipose tissue^14^. In addition to an increased availability of plasma free fatty acids, delivery of fatty acids to exercising muscles is significantly increased at those intensities^14^. In contrast, the decline in fatty acid oxidation during high intensity exercise is associated with reduced plasma fatty acid concentrations and increased accumulation of intramuscular fatty acids^14^^, ^^55^^, ^^56^. Previous studies have demonstrated that the decline in fatty acid oxidation during high intensity exercise is neither attributable to a failure of adipose tissue to deliver fatty acids nor to a decline in lipolysis of triglycerides^9^^, ^^10^. Instead, an inability of muscle cells to utilize fatty acids coupled with reduced plasma fatty acid concentrations with high exercise intensities has been suggested^9^^, ^^10^.

Evidence suggests that the increment of fatty acid transport across the muscle membrane is associated with fatty acid oxidation during low to moderately intense exercise^57^. However, the association between fatty acid uptake and fatty acid oxidation is disrupted when exercise intensity is further increased^57^. Raney and colleague^57^ found that fatty acid oxidation is reduced even if fatty acid uptake is elevated during high intensity exercise using a perfused rat hind limb model. These results support the notion that fatty acid transport across the membrane is not the major contributing factor that limits fatty acid oxidation when exercise is switched into high intensity.

The contribution of intramuscular fatty acids released by hydrolysis of intramuscular triglycerides to regulate fatty acid oxidation varies depending on the intensity of exercise^58^. Brechtel et al.^59^ demonstrated that moderately intense exercise (60–70% of peak VO_2_) decreases intramuscular triglyceride content in both the soleus and tibialis anterior muscles, whereas intramuscular triglyceride content was not altered with high intensity exercise. Similarly, no changes in intramuscular triglyceride content with high intensity exercise were observed in other studies^60^^, ^^61^, indicating that the amount of fatty acids taken up by muscles is increased during moderately intense exercise, but not during high intensity exercise. During moderately intense exercise, enhanced hydrolysis of intramuscular triglycerides appears to be involved in recruiting both hormone sensitive lipase and triglyceride lipase to the lipid droplet, attributable to a calcium- and catecholamine-mediated event^62^. However, other studies have demonstrated that hormone sensitive lipase activity is not associated with exercise intensity^63^^, ^^64^.

## Combined Effects of Exercise with Phytochemicals on Fatty Acid Oxidation

It is possible that exercise in combination with dietary phytochemical intake results in an additive effect on fatty acid oxidation because exercise boosts fatty acid oxidation and certain phytochemicals also potently stimulate fatty acid oxidation over short periods. Few studies have focused on the combined effects of exercise and phytochemicals on fatty acid oxidation.

Some phytochemicals have an additive effect on fat oxidation when combined with moderately intense exercise (Table 1). Gutierrez-Hellin et al.^48^ examined the efficacy of an acute dose of *p*-synephrine (3 mg/kg) and exercise on fatty acid oxidation using a randomized, double-blind, and counterbalanced experimental design. They found that *p*-synephrine ingestion combined with moderately intense exercise (40%~80% VO_2max_) significantly improved maximal fatty acid oxidation rate over that of the placebo treatment. In agreement with this finding, catechin-rich green tea extract supplementation (340 mg polyphenols and 136 mg EGCG) in combination with moderately intense steady-state exercise (30 min cycling at 60% Wmax) also had an additive effect on the rate of fatty acid oxidation and lipolysis^28^. However, a study of anthocyanins only showed an accumulative effect when combined with a specific exercise intensity^39^. Cook and colleagues^39^ recently investigated the effects of anthocyanin-rich New Zealand blackcurrants on fatty acid oxidation at different exercise intensities (45%, 55%, and 65% VO_2max_). They found that 7 days of ingesting New Zealand blackcurrants (105 mg anthocyanin/day) elevated fatty acid oxidation during exercise at 65% maximal intensity only. The reasons for this discrepancy were not disclosed; however, multiple factors such as the nature of phytochemicals, bioavailability, and action on multiple molecular targets might be involved in the differing beneficial effects.

**Table 1.** Additive effects of some phytochemicals with exercise on fatty acid oxidation.

Study	Subjects	Phytochemicals (amount, duration)	Exercise	Results
Venables et al. (28)	12 healthy male	Polyphenols(340mg), EGCG(136mg)	30min, 60% of Wmax exercise	↑ fat oxidation rate ↑ fat utilization in total energy expenditure
Gahreman et al. (29)	14 healthy female	Polyphenols(187.5mg), EGCG(125mg)	20min of interval sprinting exercise	↓ RER ↑ plasma glycerol ↑ epinephrine
Cook et al. (39)	14 healthy male	Anthocyanin (105mg/day, 7days)	30min, 45%, 55%, 65% of VO_2max_ cycling exercise	↑ fat oxidation rate (at 65% of VO_2max_) ↓ RER
Gutierrez-Hellin et al. (48)	18 young and healthy people	P-synephrine (3mg/kg, 60min before	40~100% of VO2max cycling exerc exercise	↑ fat oxidation (at 40~80% of VO_2max_) ↑ maximal fat oxidation
Dolinsky et al. (71)	50 8-week old male Wistar rats	Resveratrol (4g/kg, ~146mg/kg/day)	12 weeks, 10~20m/min treadmill exercise	↓ RER ↑ fat oxidation ↑ fat running time ↑ running distance

Interestingly, total energy expenditure did not change after supplementation with *p*-synephrine, catechins, or anthocyanins during moderately intense exercise, indicating that supplementation with those phytochemicals may modify substrate utilization at moderate exercise intensities (approximately 40%–80% VO_2max_)^28^^, ^^39^^, ^^48^. However, it was impossible to determine from those studies if the lack of differences in total energy expenditure during exercise was attributable to the *p*-synephrine, catechin, or anthocyanin contained in the supplements or a lack of other active substances such as caffeine, which is often included in that type of supplement and is well-known to be thermogenic^48^.

One study showed an additive effect with high intensity exercise and catechins. Gahreman et al.^29^ investigated the combined effects of acute high intensity exercise (i.e., intermittent sprinting) and catechin-containing green tea supplementation (187.5 mg polyphenols and 125 mg EGCG) on fatty acid oxidation. Using a double-blinded crossover design with two exercise sessions and either catechin or placebo, the researchers found that fatty acid oxidation was significantly higher (24–29 %) in the exercise with catechin group than that of the exercise with placebo group at rest, during exercise, and post-exercise.

**Figure 1.**
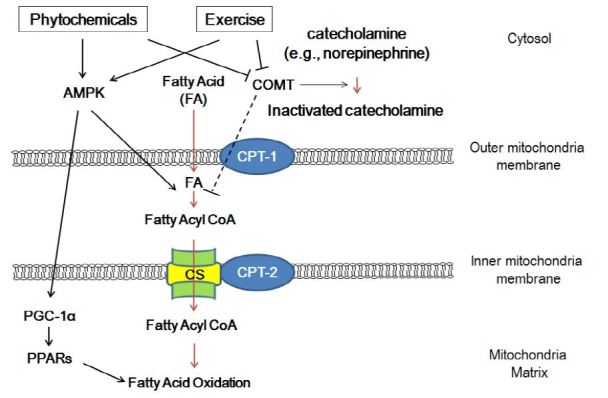
Potential regulatory pathways responsible for fatty acid oxidation with phytochemicals and exercise. AMPK, AMP-activated protein kinase; COMT, catechol-O-methyltransferase; CS, carnitine shuttle; CPT-1, carnitine palmitoyltransferase-1; CPT-2, carnitine palmitoyltransferase- 2; PGC-1α, peroxisome proliferator-activated receptor gamma coactivator-1; PPARs, peroxisome proliferator-activated receptors

Molecular and cellular mechanisms responsible for the additive effects of exercise with phytochemicals on fatty acid oxidation are unknown; however, some studies have suggested possible underlying mechanisms (Figure 1). First, increased fat oxidation with either supplementation of phytochemicals or exercise may be in part through inhibition of catechol-O-methyltransferase^65^^-^^67^. Catechol-O-methyltransferase is an enzyme that degrades norepinephrine, and its inhibition possibly prolongs the action of norepinephrine, which stimulates lipolysis and drives the breakdown of intramuscular triglycerides^68^. Second, it has been previously suggested that the polyphenol component of phytochemicals has a weak binding affinity to the α (α1 and α2) and β subunits (β1 and β2) of adrenergic receptors^69^. In contrast, polyphenols have a strong binding affinity to the β3 subunits of adrenergic receptors, which may account for the increase in fatty acid oxidation during exercise through epinephrine and norepinephrine-induced activation of adenylate cyclase and lipolysis^70^.

## CONCLUSION

It can be concluded that, to date, clinical trials investigating an additive effect of phytochemicals and exercise on fatty acid oxidation are limited. Some studies examining phytochemicals combined with exercise, as reviewed above, show potentially promising and additive effects on fatty acid oxidation. However, for most plant-based chemical compounds and extracts, more studies are needed to determine optimal doses as well as efficacy involved with long-term use with different types of exercise to enhance fatty acid oxidation. From a practical standpoint, the additive effects of combining phytochemicals with exercise on fatty acid oxidation can provide new insights for developing strategies that facilitate fat loss and improve weight management in obese patients.
